# Effect of Luteolin and Apigenin on the Expression of Oct-4, Sox2, and c-Myc in Dental Pulp Cells with *In Vitro* Culture

**DOI:** 10.1155/2015/534952

**Published:** 2015-02-26

**Authors:** Lu Liu, Zhengjun Peng, Zezhen Xu, Xi Wei

**Affiliations:** Operative Dentistry and Endodontics, Guanghua School of Stomatology, Affiliated Stomatological Hospital, Guangdong Province Key Laboratory of Stomatology, Sun Yat-Sen University, Guangzhou, Guangdong 510055, China

## Abstract

*Introduction*. Dental pulp cells (DPCs) are promising cell source for dental tissue regeneration. Recently, small molecules which optimize microenvironment or activate the reprogramming network provide a new way to enhance the pluripotency. Two promising bioflavonoids luteolin and apigenin were reported to enhance reprogramming efficiency in mouse embryonic fibroblast (MEF). However, their effect and underlying mechanism in cell fate determination of human DPCs remain unclear. *Methods*. To elucidate the effect of luteolin and apigenin on the cell fate determination of DPCs, we explored the cell proliferation, cell cycle, senescence, apoptosis, expression of pluripotency markers Oct-4, Sox2, and c-Myc, and multilineage differentiation capability of DPCs with luteolin or apigenin treatment. *Results*. We demonstrated that luteolin and apigenin inhibited cell proliferation, arrested DPCs in G2/M and S phase, and upregulated PI value and apoptosis. Moreover, luteolin and apigenin increased telomerase activity, maintained DPCs in a presenescent state, and activated the expression of Oct-4, Sox2, and c-Myc at a dose- and time-dependent pattern in DPCs even at late passages, albeit repressed lineage-specific differentiation. *Conclusions*. Addition of luteolin and apigenin in the culture medium might provide an effective way to maintain DPCs in an undifferentiated stage and inhibit lineage-specific differentiation.

## 1. Introduction

Dental pulp cells (DPCs) with self-renewal, colony forming efficiency, and multilineage differentiation capability are promising cell source for dental tissue regeneration [1, 2]. It is verified that DPCs were capable of differentiating into odontoblasts, adipocytes, chondrocytes, and so forth [1]. Since DPCs are easily obtained from extracted teeth, they may be ideal cell resource to repair injured tooth structures. However, the potential application of DPCs in dental regeneration is limited by loss of stem cell characteristics in* in vitro* culture conditions [3].

Mesenchymal stem cells from dental tissues are able to be reprogrammed to induced pluripotent stem cells (iPSCs) with embryonic stem cells (ESCs) like characteristics by introducing transcription factors Oct-4/Sox2/Klf4/c-Myc or Oct-4/Sox2/Nanog/Lin28. Thus, the transcription factors Oct-4, Sox2, and c-Myc are closely correlated with pluripotency and reprogramming [4]. Recent studies reported that small molecules increased the expression of STRO-1, Nanog, Oct-4, and Sox2 and decreased cell proliferation and odonto/osteogenic, adipogenic, and neurogenic lineages differentiation through Ras-GAP-, ERK1/2-, and mTOR-signaling pathways. Small molecules might provide a new way to enhance the immature state and maintain the potential capability of dental derived cells in tissue engineering through optimizing microenvironment [5]. Therefore, improving culture condition through adding growth factors to culture solution might be a simple and effective way of optimizing microenvironment and delivering signals to cells. Luteolin and apigenin, two important flavonoids, possess antiproliferation, proapoptosis, antiangiogenesis, antitumor, and anti-inflammatory properties [6, 7]. They are able to counteract oxidative mechanisms and suppress cell growth of prostate cancer through inhibition of insulin-like growth factor-I receptor, Akt signaling, cell cycle arrest, and induction of cell apoptosis [6, 7]. Notably, luteolin and apigenin are reported to enhance reprogramming efficiency through the upregulation of E-cadherin, which could replace Oct-4 during iPSC generation [8]. However, to date, the specific role of luteolin and apigenin in regulating pluripotency and multilineage differentiation capability of DPCs remains unknown.

Our previous study revealed that Oct-4, Sox2, and c-Myc maintained nucleus location and relatively high mRNA expression even at late passages in periodontal ligament cells (PDLCs) with rhBMP4 induction, indicating that small molecules may provide a suitable microenvironment to maintain PDLCs in an undifferentiated stage [9]. These results implied that chemical approaches may play essential roles in the regulation of cell fate determination and pluripotency, which will shed light on the potential application of DPCs in dental regeneration. Therefore, in the present study, we investigated the effect of luteolin and apigenin on cell proliferation, apoptosis, cell cycle, senescence, expression of pluripotency markers (Oct-4, Sox2, and c-Myc), and multilineage differentiation capability of DPCs, to demonstrate the essential role luteolin and apigenin played in cell fate determination of dental derived cells.

## 2. Materials and Methods

### 2.1. Isolation and Expansion of Human DPCs

Normal human premolars and impact third molars were extracted from healthy young adults (12–28 years) undergoing orthodontic treatment at the Department of Oral and Maxillofacial Surgery, the Affiliate Stomatology Hospital of Sun Yat-Sen University; informed consent was obtained from each patient. The protocols were approved by the University Ethic Committee. DPCs were obtained from dental pulp tissue by explant culture as previously described [10]. DPCs were cultured in Dulbecco's modified Eagle medium with low glucose (DMEM-LG, Invitrogen, CA, USA) supplemented with 10% fetal bovine serum (FBS, HyClone, UT, USA), 10 U/mL penicillin G, and 10 mg/mL streptomycin (Invitrogen, CA, USA). DPCs were incubated at 37°C in 5% CO_2_. Luteolin (Sigma-Aldrich, MO, USA) and apigenin (Sigma-Aldrich, MO, USA) were dissolved in dimethyl sulfoxide (DMSO, Invitrogen, CA, USA) and diluted in medium for cell culture. DPCs were serum-deprived for 24 h before induction with luteolin and apigenin at the concentrations of 0, 1 *μ*mol/L, 5 *μ*mol/L, and 10 *μ*mol/L. And the incubation was maintained for 0, 3, and 5 days. The medium was changed every 3 days.

### 2.2. Quantitative Real-Time Reverse-Transcription Polymerase Chain Reaction and Western Blot

To examine the dose- and time-dependent effect of luteolin/apigenin on the expression of Oct-4, Sox2, and c-Myc in DPCs, total RNA was isolated from DPCs at passage 3 treated with luteolin/apigenin at various concentrations (1, 5, and 10 *μ*mol/L) for 0, 3, and 5 d using Trizol reagent (Invitrogen, CA, USA) following the manufacturer's protocol. The concentration and quality of RNA samples were measured with spectrophotometers and gel electrophoresis. First-strand cDNA was synthesized from 1 *µ*g of total RNA using SuperScript III (Invitrogen, CA, USA) in a total volume of 20 *μ*L. 2.5 *μ*L of the reaction mixture was incubated with 2X SYBR Green I Master Mix (Applied Biosystems, NY, USA) in a total volume of 25 *μ*L. Primers used for detection were listed in Table 1. The conditions for PCR were as follows: 95°C for 10 min for activation, followed by 40 cycles of denaturation at 95°C for 15 s each, and finally, primer extension at 60°C for 1 min. Quantifications of* Oct-4*,* Sox2*,* c-Myc*, and* 18s* mRNA were performed using an ABI Prism 7000 sequence detection system (Applied Biosystems, Foster City, CA, USA). Each plate contained* 18s* as housekeeping gene to normalize the PCR data. All experiments were repeated three times from three separate samples. Raw data were acquired and processed to calculate the threshold cycle (Ct) value and relative gene expression values. Delta delta Ct method was performed to analyze the result.

To further investigate the effect of luteolin/apigenin on the expression of Oct-4, Sox2, and c-Myc in DPCs at various passages, quantitative real-time PCR and western blot were performed as mentioned above and described previously [11]. Briefly, the total protein was obtained from DPCs at passages 3 and 7 with/without luteolin/apigenin treatment and measured by a Bio-Rad Coomassie Blue protein assay (Bio-Rad Laboratories, Richmond, CA, USA). Twenty micrograms of protein were diluted by 10% bromophenol blue and boiled before being separated by sodium dodecyl sulfate-polyacrylamide gel electrophoresis (SDS-PAGE) and transferred to a nitrocellulose membrane. The membrane was blocked in 5% low-fat milk at room temperature for 1 h, rinsed, and incubated with mouse monoclonal antibodies against human Oct-4 (1 : 100; Chemicon MA, USA), Sox2 (1 : 50; R&D system, MN, USA), c-Myc (1 : 50; Santa Cruz, CA, USA), or human *β*-actin (1 : 1000 dilution, Santa Cruz, CA, USA) overnight at 4°C. After washing, the membrane was incubated with the HRP-conjugated secondary antibody (1 : 5000 dilution, Jackson ImmunoResearch Laboratories, PA, USA) at room temperature for 1 h. Immunoreactive proteins were then visualized by incubating membrane with electrogenerated chemiluminescence plus detection agents (GE Healthcare, NJ, USA).

### 2.3. Cell Counting Kit 8 (CCK8) Assay for Cell Proliferation

Luteolin (Sigma-Aldrich, MO, USA) and apigenin (Sigma-Aldrich, MO, USA) at the optimum concentration of 10 *μ*g/mL, indicated by result of real-time PCR and western blot mentioned above, were added to the culture medium throughout the suspension period. Cells cultured in normal medium served as control. The culture of DPCs was serum-deprived for 24 h prior to the induction. A total of 10^4^ cells per well were plated in 96-well plates and cell proliferation of DPCs was evaluated using the CCK8 (Dojindo, Tokyo, Japan) according to manufacturer's instructions. Briefly, 10 *μ*L of CCK8 solution was added to the culture medium and incubated for additional 3 h. The absorbance was determined at 450 nm wave length.

### 2.4. Flow Cytometry for Cell Cycle and Apoptosis

The culture of DPCs was serum-deprived for 24 h prior to the induction. 1 × 10^5^ DPCs with/without luteolin and apigenin treatment were harvested by trypsinization, washed twice in cold PBS, and fixed in 70% alcohol for 30 min on ice. After washing in cold PBS three times, cells were incubated with 0.5% propidium iodinate (PI) for 30 min at 4°C. Cells were analyzed using a FACSCalibur flow cytometer (BD Biosciences, San Jose, CA, USA). Data was analyzed using FCS Express software.

### 2.5. Beta-Galactosidase Staining for Cell Senescence and Telomerase Activity

Cytochemical staining for the senescence-associated b-galactosidase assay was performed by seeding DPCs from passages 1, 3, 5, and 7 with/without luteolin/apigenin treatment at the cell density of 1 × 10^3^ cells/well in a 24-well plate. The cells were allowed to attach overnight, washed with PBS, fixed, and incubated overnight at 37°C with an X-gal chromogenic substrate at pH 6.0 according to the protocol provided by the b-galactosidase staining kit (Cell Signaling Technology, MA, USA). The images of cell morphology were captured under an inverted light microscope. Telomerase activity in DPCs from passages 1, 3, 5, and 7 with/without luteolin/apigenin treatment was detected by using a quantitative telomerase detection kit (Allied Biotech, MD, USA) according to the manufacturer's protocol.

### 2.6. Effect of Luteolin and Apigenin on Odontogenic, Adipogenic, and Chondrogenic Differentiation of DPCs

For odontogenic differentiation, DPCs at passage 3 with/without luteolin and apigenin induction were odontogenically inducted in medium containing 10 mM *β*-glycerophosphate (Sigma-Aldrich, MO, USA), 50 *μ*M ascorbic acid (Sigma-Aldrich, MO, USA), and 100 nM dexamethasone (Sigma-Aldrich, MO, USA) for 21 d. The expression of DSPP was detected by immunofluorescent staining as previously described [10]. Briefly, DPCs were cultured in chamber slides (Nunc, NY, USA) and fixed with 3% paraformaldehyde for 15 min. The slides were rinsed in PBS 3 times for 5 min, respectively, then permeabilized with 0.1% Triton for 20 min, and incubated with 10% swine serum for 1 h. Slides were transferred to a humidified chamber and stained with DSPP antibody (1 : 400 dilution; Chemicon, MA, USA) overnight at 4°C. Samples were washed 3 times in PBS and incubated with a fluorochrome-labeled secondary antibody (1 : 100 dilution; Invitrogen, NY, USA) for 3 h. The sections were thoroughly washed in PBS and mounted. PBS was used instead of the primary antibody as control. The images were captured under microscope (Axiovert, Zeiss, Germany). The gene expression of* ALP* and* DSPP* was evaluated by real-time PCR as mentioned above. Primers used for detection are listed in Table 2.

For adipogenic differentiation, DPCs were incubated in the adipogenic induction medium consisting of 0.5 mM 3-isobutyl-1-methylxanthine (IBMX; Sigma-Aldrich, MO, USA), 10 *μ*g/mL insulin (GIBCO-BRL Life Technologies, Breda, Netherlands), 1 mM dexamethasone (Sigma-Aldrich, MO, USA), 100 *μ*M indomethacin (Sigma-Aldrich, MO, USA), and 15% FBS in *α*-MEM, followed by the adipogenic maintenance medium consisting solely of 10 *μ*g/mL insulin and 15% FBS. Adipogenic differentiation was detected by immunofluorescent staining of LPL and the mRNAs expression of* LPL *and* PPAR*γ*2* by real-time PCR after 21 d of adipogenic induction. DPCs were stained with LPL antibody (1 : 400 dilution; Chemicon, MA, USA). LPL expression in DPCs was investigated under a fluorescence microscope (Axiovert, Zeiss, Germany).

For chondrogenic differentiation, DPCs were chondrogenically inducted by culturing in high cell density through pelletation (2 × 10^5^ cells per pellet) in 500 *μ*L chondrogenic differentiation medium. Serum-free chondrogenic differentiation medium consisted of high glucose DMEM supplemented with 10 ng/mL of transforming growth factor-*β*3 (TGF-*β*3; R&D Systems, MN, USA), 10 nM dexamethasone (Sigma-Aldrich, MO, USA), 50 mg/mL of ascorbic acid (Sigma-Aldrich, MO, USA), 10 mg/mL of sodium pyruvate (Sigma-Aldrich, MO, USA), 10 mg/mL of proline (Sigma-Aldrich, MO, USA), and an insulin-transferrin-selenium supplement. Pellets were allowed to differentiate under 3-dimensional conditions in 15 mL centrifuge tubes at 2% or 20% O_2_ tension. After 21 days of chondrogenic differentiation, the pellets were fixed with 4% PFA and embedded in paraffin. Expression of collagen type II was examined by immunofluorescent staining and real-time PCR. DPCs were stained with collagen type II antibody (1 : 400 dilution; Chemicon, MA, USA).

### 2.7. Statistical Analysis

All experiments were repeated at least three times. The SPSS19.0 software package (SPSS Inc, Chicago, IL, USA) was used for the statistical tests. All the data were analysed using one-way ANOVA analysis and Student's *t*-test. The difference was considered as being of statistical significance at *P* < 0.05.

## 3. Results

### 3.1. The Dose- and Time-Dependent Effect of Luteolin and Apigenin on the Expression of* Oct-4, Sox2*, and* c-Myc* in DPCs

**Figure 1:**
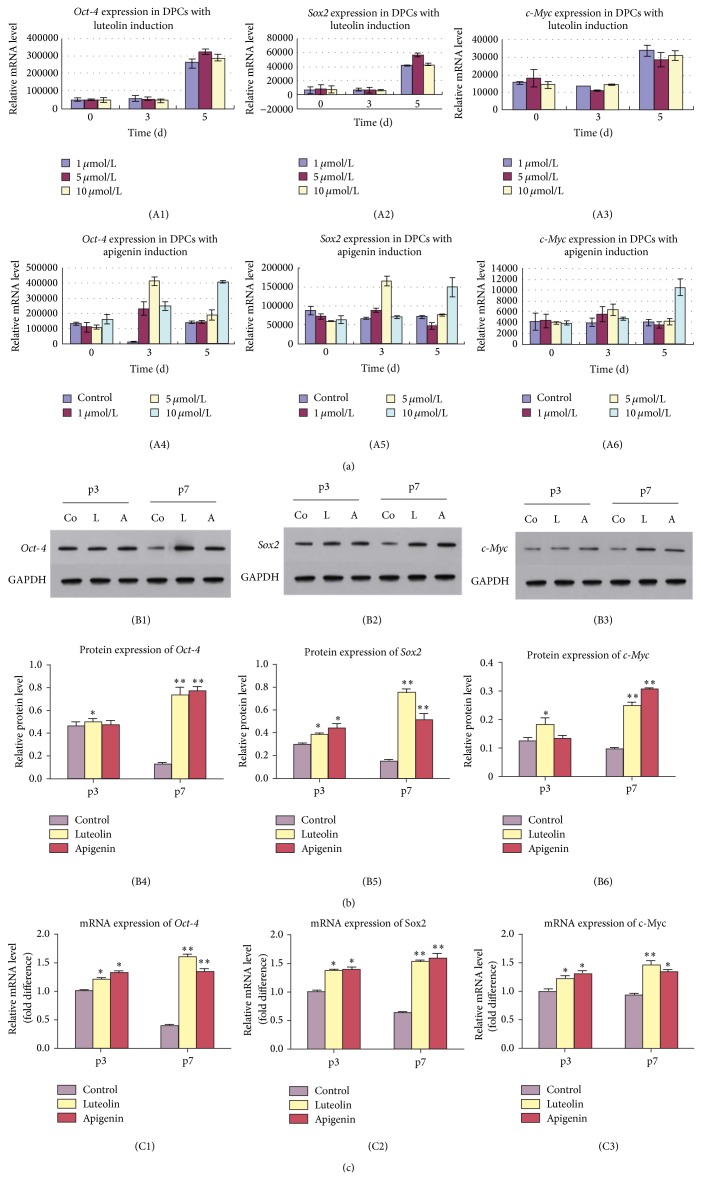
Effect of luteolin and apigenin on the expression of Oct-4, Sox2, and c-Myc in DPCs. Real-time PCR showed that mRNA expression of* Oct-4 *((A1), (A4)),* Sox2* ((A2), (A5)), and* c-Myc* ((A3), (A6)) in DPCs with luteolin ((A1)–(A3)) and apigenin ((A4)–(A6)) treatment increased at a time- and dose-dependent pattern. Expression of* Oct-4* (A1),* Sox2* (A2), and* c-Myc* (A3) was significantly upregulated in DPCs after being treated with luteolin at the concentrations of 1, 5, and 10 *μ*mol/L at 5 d (^*^ *P* < 0.05, ^**^ *P* < 0.001), albeit they did not show any difference at 0 and 3 d (*P* > 0.05). After 3 d treatment, expression of* Oct-4* (A4) was significantly upregulated in DPCs with apigenin induction at the concentrations of 1, 5, and 10 *μ*mol/L,* Sox2 *(A5) was significantly upregulated in DPCs with apigenin induction at the concentrations of 1, 5 *μ*mol/L, and* c-Myc* (A6) was significantly upregulated in DPCs with apigenin induction at the concentration of 5 *μ*mol/L (^*^ *P* < 0.05, ^**^ *P* < 0.001). After 5 d treatment, expression of* Oct-4* (A4),* Sox2* (A5), and* c-Myc* (A6) was significantly upregulated in DPCs with apigenin induction at the concentration of 10 *μ*mol/L (^*^ *P* < 0.05,^**^ *P* < 0.001). The result of real-time PCR indicated that the expression of* Oct-4* (C1),* Sox2* (C2), and* c-Myc* (C3) was significantly upregulated in DPCs at both passages 3 and 7 with luteolin and apigenin induction (^*^ *P* < 0.05, ^**^ *P* < 0.001). The protein expression of Oct-4 ((B1), (B4)), Sox2 ((B2), (B5)), and c-Myc ((B3), (B6)) was significantly upregulated in DPCs at passages 3 and 7 with luteolin induction (^*^ *P* < 0.05, ^**^ *P* < 0.001), which was similar to the expression pattern in DPCs at passage 7 with apigenin induction (^*^ *P* < 0.05, ^**^ *P* < 0.001). Whereas in DPCs, at passage 3 with apigenin induction, only Sox2 showed significant upregulation (^*^ *P* < 0.05), Oct-4 and c-Myc did not show any significant difference with the control group (*P* > 0.05).

DPCs with/without luteolin and apigenin treatment were examined for* Oct-4, Sox2*, and* c-Myc* mRNA expression by real-time PCR (Figure 1). The mRNA expression of* Oct-4* (Figures 1(A1) and 1(A4)),* Sox2* (Figures 1(A2) and 1(A5)), and* c-Myc* (Figures 1(A3) and 1(A6)) in DPCs with luteolin (Figures 1(A1)–1(A3)) or apigenin (Figures 1(A4)–1(A6)) treatment increased at a time- and dose-dependent pattern. Expression of* Oct-4* (Figure 1(A1)),* Sox2 *(Figure 1(A2)), and* c-Myc* (Figure 1(A3)) was significantly upregulated in DPCs after being treated with luteolin at the concentrations of 1, 5, and 10 *μ*mol/L at 5 d (^*^ *P* < 0.05, ^**^ *P* < 0.001), albeit they did not show any difference at 0 and 3 d (*P* > 0.05). However, the expression of* Oct-4, Sox2* and* c-Myc *in DPCs with apigenin treatment showed a different pattern. After 3 d treatment, expression of* Oct-4* (Figure 1(A4)) was significantly upregulated in DPCs with apigenin induction at the concentrations of 1, 5, and 10 *μ*mol/L,* Sox2* (Figure 1(A5)) was significantly upregulated in DPCs with apigenin induction at the concentration of 1, 5 *μ*mol/L, and* c-Myc* (Figure 1(A6)) was significantly upregulated in DPCs with apigenin induction at the concentration of 5 *μ*mol/L (^*^ *P* < 0.05, ^**^ *P* < 0.001). After 5 d treatment, expression of* Oct-4* (Figure 1(A4)),* Sox2* (Figure 1(A5)), and* c-Myc* (Figure 1(A6)) was significantly upregulated in DPCs with apigenin induction at the concentration of 10 *μ*mol/L (^*^ *P* < 0.05, ^**^ *P* < 0.001). Therefore, 10 *μ*mol/L and 5 d of induction were chosen as the optimized concentration and time for induction in the following experiment.

### 3.2. Effect of Luteolin and Apigenin on the Expression of Oct-4, Sox2, and c-Myc in DPCs at Various Passages

The result of real-time PCR indicated that expression of* Oct-4* (Figure 1(C1)),* Sox2* (Figure 1(C2)), and* c-Myc* (Figure 1(C3)) was significantly upregulated in DPCs at both passages 3 and 7 with luteolin and apigenin induction (^*^ *P* < 0.05, ^**^ *P* < 0.001). This result was confirmed by western blot. The protein expression of Oct-4 (Figures 1(B1) and 1(B4)), Sox2 (Figures 1(B2) and 1(B5)), and c-Myc (Figures 1(B3) and 1(B6)) was significantly upregulated in DPCs at passages 3 and 7 with luteolin induction (^*^ *P* < 0.05, ^**^ *P* < 0.001), which was similar to the expression pattern in DPCs at passage 7 with apigenin induction (^*^ *P* < 0.05, ^**^ *P* < 0.001). Whereas in DPCs at passage 3 with apigenin induction only Sox2 showed significant upregulation (^*^ *P* < 0.05), Oct-4 and c-Myc did not show any significant difference with the control group (*P* > 0.05).

### 3.3. Effects of Luteolin and Apigenin on Cell Proliferation, Cell Cycle, and Apoptosis of DPCs

**Figure 2:**
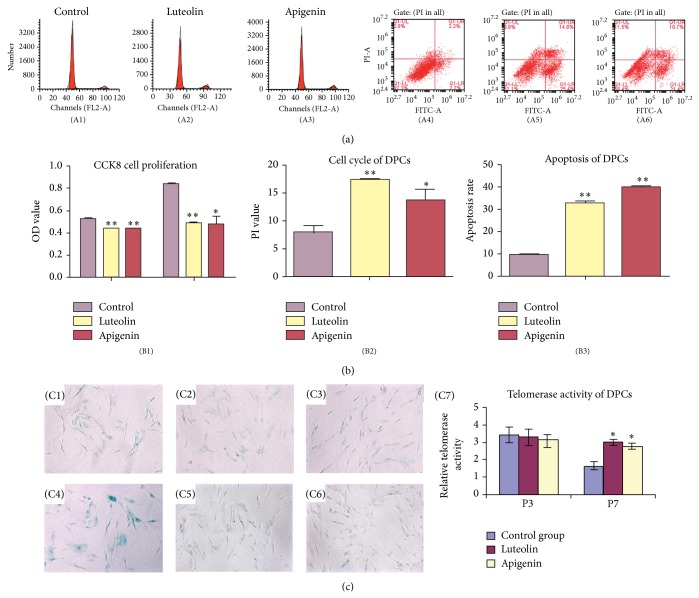
Effect of luteolin and apigenin on cell proliferation, cell cycle, apoptosis, cell senescence, and telomerase activity of DPCs. The cell cycle of DPCs with luteolin or apigenin induction was detected by fluorescence activated cell sorter (FACS) ((A1)–(A6)). The percentage of propidium iodinate (PI) = (S + G2/M)% and apoptosis of DPCs was significantly upregulated with luteolin or apigenin treatment compared with control group ((B2), (B3); ^*^ *P* < 0.05, ^**^ *P* < 0.001), whereas CCK8 revealed that cell proliferation rate was significantly restrained in DPCs with luteolin or apigenin treatment ((B1); ^*^ *P* < 0.05, ^**^ *P* < 0.001). The result of SA-b-gal revealed that DPCs at passage 3 ((C1)–(C3)) with luteolin (C2)/apigenin (C3) induction and the control group (C1) did not show any obvious blue staining (×100), whereas DPCs at passage 7 ((C4)–(C6)) without induction (control group (C4)) showed intense blue color. DPCs at passage 7 with luteolin (C5) or apigenin (C6) induction revealed weak blue staining, not as intense as the control group at passage 7 ((C4), ×100). The telomerase activity of DPCs at passage 3 with/without luteolin or apigenin induction showed no significant difference ((C7), *P* > 0.05), albeit DPCs at passage 7 with luteolin or apigenin induction revealed significantly higher telomerase activity than the control group at passage 7 (^*^ *P* < 0.05).

The effect of luteolin and apigenin on cell proliferation, cell cycle, and apoptosis of DPCs was investigated. As shown in Figure 2(b), treatment with luteolin and apigenin significantly restrained the cell proliferation of DPCs (Figure 2(B1); ^*^ *P* < 0.05, ^**^ *P* < 0.001), whereas the apoptosis of DPCs was significantly upregulated with luteolin and apigenin induction (Figure 2(B3); ^*^ *P* < 0.05, ^**^ *P* < 0.001). Furthermore, luteolin and apigenin arrested DPCs in G2/M and S phase of the cell cycle, demonstrated by significant upregulation of the percentage of cells in G2/M and S phase, with a significant downregulation in G0/G1 phase (Figures 2(A1)–2(A6), Table 3; ^*^ *P* < 0.05, ^**^ *P* < 0.001). Apoptotic sub-G0/G1 cells were not detected. The percentages of PI = (S + G2/M)% in luteolin and apigenin treated DPCs were significantly higher than the control group (Figure 2(B2), Table 3; ^*^ *P* < 0.05, ^**^ *P* < 0.001). These data indicated that luteolin and apigenin inhibited cell proliferation, arrested DPCs in G2/M and S phase, and upregulated PI value and apoptosis.

### 3.4. Effect of Luteolin and Apigenin on the Cell Senescence and Telomerase Activity of DPCs at Various Passages

Senescence-associated b-galactosidase is caused by upregulated lysosomal activities and altered cytosolic pH, which are upregulated with senescence and aging. To elucidate the effect of luteolin and apigenin on replicative senescence state of DPCs, the senescence-associated b-galactosidase activity (SA-b-gal) was evaluated. DPCs from passages 1, 3, 5, and 7 with/without luteolin/apigenin treatment were detected, albeit only the representative results of passages 3 and 7 were presented. The result revealed that DPCs at passage 3 with luteolin (Figure 2(C2))/apigenin (Figure 2(C3)) induction and the control group (Figure 2(C1)) did not show any obvious blue staining. DPCs at passage 7 without induction (control group, Figure 2(C4)) showed intense blue color, albeit DPCs at passage 7 with luteolin (Figure 2(C5)) or apigenin (Figure 2(C6)) induction revealed weak blue staining, not as intense as the control group at passage 7. Similarly, there is no difference of the telomerase activity of DPCs at passage 3 with/without luteolin or apigenin induction (Figure 2(C7), *P* > 0.05), albeit DPCs at passage 7 with luteolin or apigenin induction showed significantly higher telomerase activity than the control group at passage 7 (^*^ *P* < 0.05), which agreed with the result of b-galactosidase assay mentioned above. This result implied that luteolin and apigenin treatment significantly inhibited cell senescence and increased telomerase activity of DPCs, especially at late passages. Thus, luteolin and apigenin might be able to maintain DPCs in an undifferentiated and presenescent state.

### 3.5. Effect of Luteolin and Apigenin on the Multilineage Differentiation Capability of DPCs

**Figure 3:**
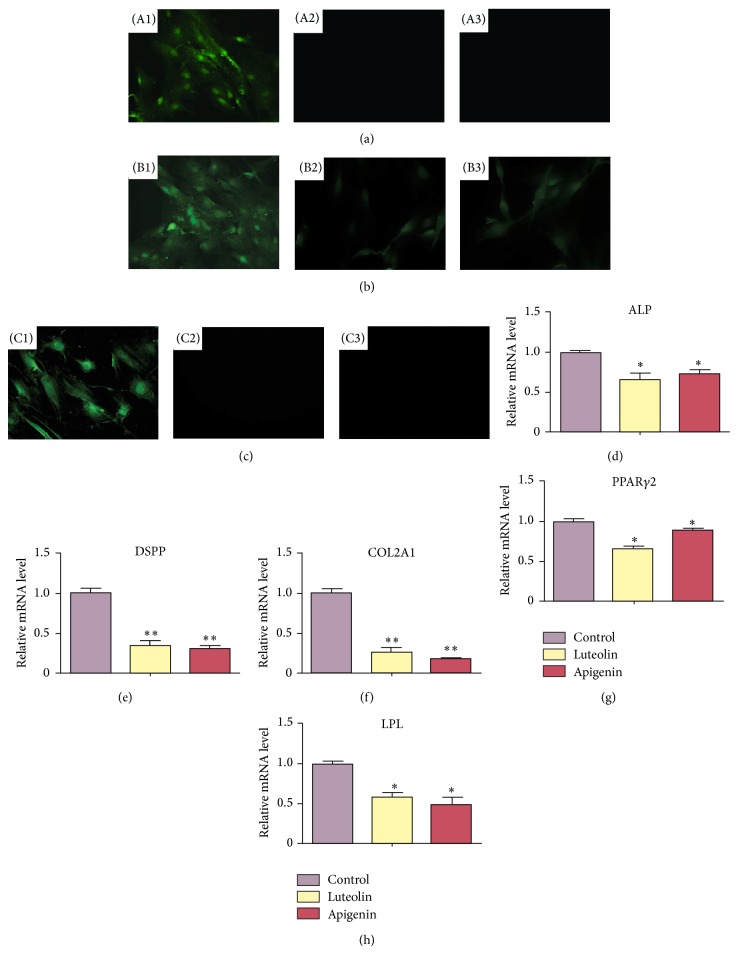
Effect of luteolin and apigenin on the multilineage differentiation capability of DPCs. The immunofluorescent staining revealed that DSPP (A1) and LPL (B1) were strongly expressed in DPCs without luteolin/apigenin treatment after 21 d of odontogenic and adipogenic induction, mainly located in the nucleus of DPCs. Collagen type II (C1) was mainly located in the cytoplasm of DPCs without luteolin/apigenin treatment after 21 d of chondrogenic induction, whereas, for the DPCs with luteolin/apigenin treatment, only LPL ((B2), (B3)) showed weak expression in the nucleus of DPCs after 21 d of adipogenic induction, albeit DSPP ((A2), (A3)) and collagen type II ((C2), (C3)) were barely found in DPCs. Real-time PCR indicated that mRNA expression of osteogenic markers (*ALP, DSPP*), adipogenic markers (*PPAR*γ*2, LPL*), and chondrogenic markers (*collagen type II*) was significantly downregulated in luteolin and apigenin groups after 21 d induction towards multilineages compared with control group ((d)–(h)).

Assay of the multilineage differentiation capability of DPCs towards odontogenic, chondrogenic, and adipogenic cell lineages showed considerable variation in culture condition treated with luteolin and apigenin. The immunofluorescent staining showed that DSPP (Figure 3(A1)) and LPL (Figure 3(B1)) were strongly expressed in DPCs without luteolin/apigenin treatment after 21 d of odontogenic and adipogenic induction, mainly located in the nucleus of DPCs. Collagen type II (Figure 3(C1)) was mainly located in the cytoplasm of DPCs without luteolin/apigenin treatment after 21 d of chondrogenic induction, whereas, for the DPCs with luteolin/apigenin treatment, only LPL (Figures 3(B2) and 3(B3)) showed weak expression in the nucleus of DPCs after adipogenic induction, albeit DSPP (Figures 3(A2) and 3(A3)) and collagen type II (Figures 3(C2) and 3(C3)) were barely found in DPCs after odontogenic and chondrogenic induction. This result was confirmed by real-time PCR, which indicated that mRNA expression of odontogenic markers (*ALP, DSPP*), adipogenic markers (*PPAR*γ*2, LPL*), and chondrogenic markers (*collagen type II*) was significantly downregulated in luteolin and apigenin groups after 3w induction towards multilineages compared with control group (Figures 3(d)–3(h)). These results indicated that the multilineage differentiation capability was inhibited in DPCs with luteolin/apigenin treatment.

## 4. Discussion

Previous studies have shown that the overexpression of Oct-4, Sox2, Klf4, and Myc (OSKM) can convert mouse fibroblasts into iPSCs with resemble global gene expression, epigenetic state, and developmental potential of mESC [12, 13]. Oct-4, Sox2, and c-Myc work cooperatively in maintaining or activating the reprogramming network during this process; thus their expression levels are assumed to be closely related to pluripotency and reprogramming capability [14]. Since the loss of pluripotency could be rejuvenated by altered extracellular microenvironment and molecules controlling endogenous signaling pathways, it is possible to enhance the pluripotency and improve cell characteristics by optimizing the culture condition with certain molecules [15–17]. It is reported that alternation of O_2_ microenvironments may regulate hESCs survival, self-renewal, and differentiation capabilities through posttranscriptional regulation of telomerase isoforms [16]. Moreover, hypoxia played a critical role in maintaining the stemness and differentiation capacity of PDLCs and DPCs through reactivation of Oct-4, Sox2, and c-Myc [17]. Therefore, simple and effective ways to improve undifferentiated state of somatic cells without integrating gene delivery methods are highly desirable.

Small molecules are able to regulate specific signaling pathways involved in chromatin remodeling, gene expression alternation, and cell biology manipulation [5, 18]. Instead of activating the “master genes” by virus transfection, pluripotency and reprogramming network can be reestablished by the application of small molecules and modulation of cell culture microenvironment. Various small molecules with the advantages of low cost, biocompatibility, and easy application have been demonstrated to enhance reprogramming efficiency and improve cell characteristics. These properties make it possible for the small molecules to provide temporal modulation of the pluripotent signals [5, 18, 19]. However, the complete chemical inducing approach requires further studies and the underlying mechanism remains unclear.

Flavonoids, polyphenolic compounds produced by plants, contribute to the prevention of heart disease, neurodegenerative diseases, diabetes, and cancer [6, 7]. Flavonoids play important roles in antioxidant effect, anti-inflammatory effect, regulation of apoptosis, and suppression of tumor related genes and DNA damage through modulation of growth factor signaling pathways [6, 7]. Antioxidant effect is one of the main biological functions of flavonoids, which contributes to chelating metal ions and inactivating free radicals [20]. The flavonoids luteolin and apigenin possess antiproliferation, proapoptosis, antiangiogenesis, antitumor, and anti-inflammatory properties [6]. These two compounds strongly dose-dependently inhibited tumor necrosis factor *β*- (TNF-*β*-) induced NF-*κ*B, IL-8, and E-selectin protein expression after stimulation with lipopolysaccharide (LPS) or TNF-*β* in endothelial cells [20, 21]. Luteolin showed synergistic antiviral effect with IFN-*β* in modulating the immune response of peripheral blood mononuclear cells [22]. Luteolin, inhibitor of cyclin-dependent kinase 9 (CDK9), can induce apoptosis in cancer cells through blocking phosphorylation of the carboxy-terminal domain of RNA polymerase II [23]. Apigenin restrained prostate carcinogenesis via TGF-*β*-activated pathways, especially the Smad2/3 and Src/FAK/Akt pathways [24].

Luteolin regulated proliferation and cell cycle transition of PC-3 cell through EGFR-tyrosine kinase independent mechanism, which arrested cells in G2/M phase, decreased the number of cells in G0/G1, and caused significant increase in apoptotic cells [25]. It is also reported that decreasing cell proliferation by removing c-Myc or adding antiproliferative compounds enhanced iPSCs generation, implying inhibiting cell proliferation and arresting cell cycle may improve reprogramming [26]. Our present study revealed that luteolin and apigenin inhibited cell proliferation, arrested DPCs in G2/M and S phase, downregulated the number of cells in G0/G1, and upregulated apoptotic cells. Moreover, additional treatment with luteolin and apigenin dramatically enhanced the expression of pluripotency and reprogramming markers Oct-4, Sox2, and c-Myc. These results agreed with the previous studies [25, 26]. Although our study did not provide direct evidence that apoptosis is occurring in the cells arrested in G2/M phase, the data indicated that luteolin and apigenin may play important roles in cell proliferation, apoptosis, and phase-specific cell cycle regulation of DPCs, albeit further studies might be required to elucidate the underlying mechanism regulating apoptosis and cell cycle.

Consistent expression of telomerase activity inhibits senescence and is responsible for the maintenance of stemness properties [27, 28]. Oct-4, Sox2, and c-Myc are closely related to pluripotency and reprogramming property [12–14]. In this study, our results revealed that luteolin and apigenin upregulated the telomerase activity, maintained DPCs in a presenescent state, and activated the expression of Oct-4, Sox2, and c-Myc in DPCs even at late passages. These results suggested that luteolin or apigenin treated microenvironment might be effective way to trigger the expression of pluripotency markers and maintain undifferentiated state of DPCs, which agreed with previous studies that luteolin and apigenin upregulated Oct-4, Sox2, and c-Myc through E-cadherin and enhanced reprogramming efficiency [8]. However, it was shown in our study that additional luteolin and apigenin in culture condition repressed lineage-specific differentiation potential of DPCs, evidenced by the real-time PCR and immunofluorescent staining. Thus, luteolin and apigenin enhanced undifferentiated state and inhibit lineage-specific differentiation of DPCs.

This study revealed that, taken together, luteolin and apigenin could enhance the expression of pluripotency markers, maintain DPCs in an undifferentiated state, and inhibit lineage-specific differentiation, which is of critical importance for the application of DPCs in dental regeneration. However, future studies are required to investigate the underlying mechanism of small molecules in the regulation of pluripotency in DPCs with* in vitro* culture.

## Figures and Tables

**Table 1:** Primer sequences used in quantitative real-time polymerase chain reaction.

Gene	Primers
*Oct-4 *	Forward: 5′-GCT CGA GAA GGA TGT GGT C-3′
	Reverse: 5′-ATC CTC TCG TTG TGC ATA GTC G-3′

*Sox2 *	Forward: 5′-GAGAACCCCAAGATGCACAAC-3′
	Reverse: 5′-CGCTTAGCCTCGTCGATGA-3′

*c-Myc *	Forward: 5′-GGCTCCTGGCAAAAGGTCA-3′
	Reverse: 5′-AGTTGTGCTGATGTGTGGAGA-3′

*18s *	Forward: 5′-CCTGGATACCGCAGCTAGGA-3′
	Reverse: 5′-GCGGCGCAATACGAATGCCCC-3′

**Table 2:** Primer sequences used in quantitative real-time polymerase chain reaction.

Gene	Primers
*ALP *	Forward: 5′-GAC TGA CCC TTC CCT CTC G-3′
	Reverse: 5′-GTG GTC AAT CCT GCC TCC T-3′

*PPAR*γ*2 *	Forward: 5′-CTT CGG AAT CAG CTC TGT GGA C-3′
	Reverse: 5′-GCA TCC TTC ACA AGC ATG GAC T-3′

*LPL *	Forward: 5′-GGG AGT TTG GCT CCA GAG TTT-3′
	Reverse: 5′-TGT GTC TTC AGG GGT CCT TAG-3′

*Col2a1 *	Forward: 5′-TCC CAG AAC ATC ACC TAC CAC T-3′
	Reverse: 5′-GGT CTT CTG TGA TCG GTA CTC G-3′

*DSPP *	Forward: 5′-ATT CCG GTT CCC CAG TTA GTA-3′
	Reverse: 5′-CTG TTG CTA GTG GTG CTG TT-3′

*18s *	Forward: 5′-CCTGGATACCGCAGCTAGGA-3′
	Reverse: 5′-GCGGCGCAATACGAATGCCCC-3′

**Table 3:** The cell cycle of DPCs in various culture conditions (mean ± SD% *N* = 3).

Groups	G0/G1	G2/M	S	PI = (S + G2/M)%
Control	91.9 ± 0.90	3.72 ± 0.05	4.29 ± 0.35	8.00
Luteolin	82.5 ± 0.20^**^	9.28 ± 1.03^**^	8.23 ± 0.53^**^	17.5^**^
Apigenin	85.5 ± 2.90^*^	8.82 ± 0.63^*^	5.68 ± 0.23^*^	14.5^*^

^*^ *P* < 0.05, ^**^ *P* < 0.001.
